# European Reference Network For Rare Vascular Diseases (VASCERN) Outcome Measures For Hereditary Haemorrhagic Telangiectasia (HHT)

**DOI:** 10.1186/s13023-018-0850-2

**Published:** 2018-08-15

**Authors:** Claire L. Shovlin, Elisabetta Buscarini, Anette D. Kjeldsen, Hans Jurgen Mager, Carlo Sabba, Freya Droege, Urban Geisthoff, Sara Ugolini, Sophie Dupuis-Girod

**Affiliations:** 1 Respiratory Medicine, and VASCERN HHT European Reference Centre, Hammersmith Hospital, Imperial College Healthcare NHS Trust, London, UK; 2 NHLI Vascular Science, Imperial College London, London, UK; 3 Gastroenterology Department and VASCERN HHT European Reference Centre, Maggiore Hospital, ASST Crema, Crema, Italy; 4 Department of Otorhinolaryngology and VASCERN HHT European Reference Centre, Odense University Hospital, University of Southern Denmark, Odense, Denmark; 5 Department of Pulmonology and VASCERN HHT European Reference Centre, St. Antonius Hospital, Koekoekslaan 1, 3435 CM Nieuwegein, the Netherlands; 6 Center for Rare Diseases, “Frugoni” Internal Medicine Unit, Interdepartmental HHT Center, Interdisciplinary Department of Medicine and VASCERN HHT European Reference Centre, University of Bari School of Medicine, Bari, Italy; 7 Department of Otorhinolaryngology and VASCERN HHT European Reference Centre, Essen University Hospital, Essen, Germany; 8 Department of Otorhinolaryngology and VASCERN HHT European Reference Centre, Fondazione Istituto di Ricovero e Cura a Carattere Scientifico (I.R.C.C.S) Policlinico San Matteo, University of Pavia, Pavia, Italy; 9 Hospices Civils de Lyon, Hôpital Femme-Mère-Enfants, Service de Génétique, and VASCERN HHT European Reference Centre/ centre de Référence pour la maladie de Rendu-Osler, F-69677 Bron, France; 10 Université de Lyon, Faculté de médecine, Université Lyon 1, F-69008 Lyon, France; 11 Present address: Department of Otorhinolaryngology, Head and Neck Surgery, University Hospital Marburg, Philipps-Universität Marburg, Marburg, Germany

**Keywords:** Anaemia, Antibiotic prophylaxis, Epistaxis, Iron deficiency, Nosebleeds, Pulmonary arteriovenous malformations, Pregnancy

## Abstract

Hereditary haemorrhagic telangiectasia (HHT) is a multisystemic vascular dysplasia that leads to nosebleeds, anaemia due to blood loss, and arteriovenous malformations (AVMs) in organs such as the lungs, liver and brain. HHT is estimated to affect 85,000 European citizens, but most health care providers have limited prior HHT exposure or training.

Outcome Measures were developed and implemented by the HHT Working Group of the European Reference Network for Rare Vascular Diseases (VASCERN), in order to maximise the number of patients receiving good care. The measures specifically target areas where optimal management reduces morbidity and mortality in HHT patients, and were designed to be robust to emerging new evidence. Thresholds are the percentage of patients in particular settings who have been recommended screening, or provided with written advice. The 5 Outcome Measures cover (1) pulmonary AVM screening; (2) written nosebleed advice, (3) assessment of iron deficiency; (4) antibiotic prophylaxis prior to dental and surgical procedures for patients with pulmonary AVMs, and (5) written advice on pregnancy. They are not a blueprint for detailed HHT management, but are suitable for all clinicians to be aware of and implement.

In summary, these 5 Outcome Measures provide metrics to identify healthcare providers of good care, and encourage care improvement by all healthcare providers.

**Electronic supplementary material:**

The online version of this article (10.1186/s13023-018-0850-2) contains supplementary material, which is available to authorized users.

## Background

Development and implementation of Outcomes Measures are an effective part of Quality and Safety Frameworks that lead to Service Improvements. More specifically, if the Outcome Measures are carefully selected, their dissemination and implementation can directly improve patient care, including that from health care providers with limited prior exposure or training on the specific disease. Therefore simple, clinical practice-based Outcome Measures are particularly important for rare multisystemic conditions.

The current statement refers to one specific rare disease, hereditary haemorrhagic telangiectasia (HHT; Online Mendelian Inheritance in Man® #187300), which is a multisystemic vascular dysplasia that leads to telangiectasia and arteriovenous malformations (AVMs) in visceral and mucocutanous vascular beds [1]. Based on a conservative population prevalence of 1 in 6000 [2–4], HHT is estimated to affect approximately 85,000 European citizens. The main goal of management is to maximise the number of affected individuals receiving safe and effective preventative strategies in order to limit the number and severity of HHT complications. The reason this is important, is that where optimal management is instituted, previously reduced life expectancy [3, 5–7] may improve to that of the general population, [8] early strokes, brain abscess, maternal deaths and migraines are prevented, [9–11] nosebleeds are reduced, [12–17] and patients receive timely treatments for iron deficiency anaemia and other complications.

HHT can be diagnosed either clinically using the Curaçao criteria [1] (Table 1), or through a molecular gene test*.* A patient with definite HHT will have at least 3 of the 4 Curaçao Criteria, or a pathogenic sequence variant in *ENG*, *ACVRL1* or *SMAD4*. Identification of pulmonary arteriovenous malformations (PAVMs) is recommended for all HHT patients because PAVMs commonly cause preventable complications in asymptomatic patients [18, 19]. Risk-benefit analyses in asymptomatic individuals are more complex for other AVMs where screening is more controversial [20], limited to specific subpopulations, or not recommended [21].

**Table 1:** The Curaçao Criteria

1. **Epistaxis**: spontaneous, recurrent nose bleeds	
2. **Telangiectases**: multiple, at characteristic sites (lips, oral cavity, fingers, nose)	
3. **Visceral lesions** such as gastrointestinal telangiectasia (with or without bleeding); pulmonary AVM; hepatic AVM; cerebral AVM; spinal AVM	
4. **Family history**: a first degree relative with HHT according to these criteria	

The HHT diagnosis is **definite** if 3 criteria are present, **possible or suspected** if 2 criteria are present, and **unlikely** if fewer than 2 criteria are present. A pathogenic (null) sequence variant in *ENG, ACVRL1* or *SMAD4* also defines definite clinical HHT according to current understanding. A negative HHT gene test does not exclude HHT unless the gene variant causing HHT has been identified in another affected family member

## Main text

Outcome Measures have been embedded within the Operational Criteria of the new European Reference Networks (ERNs) for Rare Diseases [22]. The HHT Outcome Measures were developed and implemented by the HHT Working Group of the ERN for Rare Vascular Diseases (VASCERN), [23] in order to maximise the number of patients receiving good care. Details of how the topics were selected and developed into Outcome Measures are provided in the Additional file 1.

Following on from an established diagnosis of HHT or PAVMs, the outcome measure thresholds are the percentage of adult patients in particular settings who have been recommended screening, or provided with written advice (Table 2). These will be met easily by best practice in HHT centres of excellence, but may not be achieved in general specialty care. The current manuscript refers only to the principles of management- details of management lie outside of the scope of this manuscript.

**Table 2:** HHT outcome measures

	Target Population	Estimated cases in Europe	Measure	Target threshold
Measure 1}	All HHT- clinical or molecular diagnosis	85,000	Screen for pulmonary AVMs	≥ 90%
Measure 2}			Receive nosebleed advice in writing	≥ 90%
Measure 3}			Assessment of iron deficiency *at each consultation*	≥ 70%
Measure 4	Pulmonary AVMs (+/− HHT^a^)	196,000	Receive written advice on antibiotic prophylaxis prior to dental and surgical procedures	100%
Measure 5	Pregnant women with pulmonary AVMs (+/− HHT^a^)	~ 1000	Receive written advice on PAVM/HHT pregnancies	100%

Prevalence estimates assume a European population of 510,000,000 (196,000 with HHT or PAVMs), 100,000 females with HHT or PAVMs; and an average of 1.6 children per woman accounting for 1.2 years pregnant in 84 years life expectancy. ^a^Pulmonary AVMs also occur outside HHT and are estimated to affect 1 in 2600 people [33]. HHT and non HHT-related pulmonary AVMs are currently managed in the same way

### Measure 1

#### At least 90% of definite HHT patients will have a screen for pulmonary arteriovenous malformations (PAVMs)

As stated elsewhere [18, 19], all HHT patients should be offered a screen for PAVMs in adult life. The screen and subsequent management may require cross-specialist referral. Screening should be repeated after pregnancies. Where a childhood screening test has been performed, screening should be repeated after the patient is fully grown.

HHT-associated PAVMs affect approximately 50% of HHT patients i.e. 40,000 Europeans. Recent European data suggest that untreated, 6–8% may develop a cerebral abscess (usually with fatal or life-changing consequences [24–26]), at least 10–12% an early ischaemic stroke, and 1% of pregnancies will result in maternal death. Diagnosed patients can benefit from stroke reduction strategies, particularly embolization therapy to obliterate PAVMs [9, 27, 28], antibiotic prophylaxis to prevent cerebral and visceral abscesses from silent bacteraemia (see Measure 4), and pregnancy management associated with a statistically improved survival rate in the event of a life-threatening complication [10].

A PAVM screen should be considered in all adult HHT patients. However the 90% threshold takes into account the facts that not all HHT patients choose to take up the option of PAVM screening, and there may also be competing clinical circumstances which render the discussion of screening inappropriate at the time of the clinical review. Overall, the Outcome Measure ensures that the possibility of PAVMs is remembered and addressed for all HHT patients, irrespective of their presentation pattern.

### Measure 2

#### At least 90% of definite HHT patients will have received nosebleed advice in writing

Nosebleeds (epistaxis) affect > 90% of HHT patients, may be the presenting symptom of HHT, but may not be volunteered by patients unless specifically asked. HHT nosebleeds often occur daily, lasting many minutes and even hours: It is poorly appreciated that severe recurrent HHT nosebleeds can generate acute haemodynamic compromise and/or chronic cardiac failure in addition to iron deficiency anaemia.

A nosebleed history is to be evaluated for all HHT patients. The 90% threshold for written advice takes into account the facts that not all HHT patients have nosebleeds at any given time, though these may develop in the future, and the information can be important for family members also affected by HHT.

Overall, this measure ensures that irrespective of the patient’s presentation pattern, epistaxis is not overlooked, and practical information is provided to reduce the severity of epistaxis, and its sequelae.

### Measure 3

#### At least 70% of definite HHT patients will have an assessment of iron deficiency at each consultation

Iron deficiency affects > 60% of all HHT patients (i.e. > 50,000 Europeans with HHT), and leads to anaemia (higher likelihood of blood transfusions), malaise, suboptimal immune, skeletal muscle and thyroid function; prematurity, poor maternal and perinatal outcomes in pregnancy; and impaired motor and cognitive development in children. In HHT patients, iron deficiency leads to angina and tachycardia, aggravates high output cardiac failure, and is associated with venous thromboemboli (VTE), and ischaemic strokes.

Generally, anaemia is defined by low haemoglobin, and iron deficiency is confirmed to be present when the ferritin is sub-normal [29, 30]. As stated elsewhere however [31], in HHT, not all iron deficient patients have low haemoglobin since many patients have hypoxaemia due to pulmonary AVMs and need higher than normal haemoglobin to compensate. Similarly, a normal or high ferritin does not reliably exclude iron deficiency as it is an acute phase marker and may be supra-normal when serum iron, transferrin saturation index and red cell indices such as MCV or MCH/MCHC demonstrate iron deficient pictures [30].

Iron deficiency is commonly indolent and not appreciated by the patient until after correction. This measure ensures that irrespective of the patient’s presentation pattern, iron deficiency is promptly identified, enabling optimal management.

### Measure 4

#### 100% of patients with PAVMs will have written advice on antibiotics prior to dental and surgical procedures

This measure is recommended to reduce the rate of cerebral abscess which occurred in 6–8% of referrals to current European centres, usually resulting in substantial morbidity, and health care burdens [24–26]. Despite antibiotic prophylaxis being a long-standing recommendation for PAVM patients, in 2017, only 2/25 (8%) consecutive referrals to a UK centre for PAVM embolization had been advised to use prophylactic antibiotics.

The majority of cerebral abscesses in HHT/PAVM patients are associated with periodontal microbes, and/or precipitating dental and other interventional events that normally lead to transient bacteraemias, i.e. prior to non-sterile invasive procedures (such as dental, endoscopic, and surgical). In the general population, the bacteraemias are cleared (in terms of positive cultures) within minutes in the absence of antibiotics, but prevented or resolved earlier with prior antibiotic administration. [32] Good dental care is also important.

Written management advice to all PAVM patients is anticipated to ensure patients are aware and can communicate to all relevant practitioners, to address the current situation where patients may be refused prophylaxis and go on to develop a cerebral abscess [26].

### Measure 5

#### 100% of pregnant women with PAVMs identified by CT scan/imaging will be provided with written advice on PAVM/HHT pregnancies

Although the majority of PAVM pregnancies proceed normally, there is a 1% risk of maternal death in pregnancy that can be reduced by prior awareness. Precise content will vary by country according to obstetric care pathways but may include alerts regarding potential red flag symptoms demanding immediate hospital admission (haemoptysis, sudden acute breathlessness), antibiotic prophylaxis at delivery, and management as “high-risk” pregnancies involving a multiprofessional approach.

## Conclusion

These five Outcome Measures provide metrics to identify healthcare providers of good care, encourage care improvement by all healthcare providers, and should be robust to emerging new evidence. They are not a blueprint for detailed HHT management, but are suitable for all clinicians to be aware of and implement. For the latest information, please see documents on the VASCERN website [23].

## Additional file

Additional file 1: Methodological notes. (DOCX 28 kb)

## Abbreviations

*ACVRL1*: Gene encoding the ALK-1 protein
AVM(s): Arteriovenous malformation(s)
*ENG*: Gene encoding the endoglin protein
ERNs: European Reference Networks
HHT: Hereditary haemorrhagic telangiectasia
PAVMs: Pulmonary arteriovenous malformations
*SMAD4*: Gene encoding the SMAD4 protein
VASCERN: European Reference Network for Rare Vascular Diseases
