# Supplementary material for: European Reference Network For Rare Vascular Diseases (VASCERN) Outcome Measures For Hereditary Haemorrhagic Telangiectasia (HHT)
Source: Orphanet J Rare Dis. 2018 Aug 15;13:136. doi: 10.1186/s13023-018-0850-2 (PMC6094583; doi:10.1186/s13023-018-0850-2)
Supplement: Supplementary file 1 — Methodological notes. (DOCX 28 kb) [file 13023_2018_850_MOESM1_ESM.docx]

# European Reference Network for Rare Vascular Diseases (VASCERN) Outcome Measures for Hereditary Haemorrhagic Telangiectasia (HHT)

# Shovlin CL, Buscarini E, Kjeldsen AD, Mager JJ, Sabba C, Droege F, Geisthoff U, Ugolini S, Dupuis-Girod S.

**DATA SUPPLEMENT**

**Methodological Notes:**

In April 2016, Leads from all 8 of the proposed VASCERN HHT healthcare providers (HCPs) were asked to suggest Outcome Measures which were to be embedded in the formal June 2016 submission of the ERN Network Application, and the Healthcare Providers' applications.

An initial list of 8 potential topics was included in a Survey to which all could add comments (Supplementary Table 1). The list was expanded further as a result of the survey to 14 topics related to screening or management of nosebleeds, severe bleeds, anemia, pulmonary AVMs, cerebral AVMs, hepatic AVMs, and pregnancy.

Further information was received from the European Commission emphasising first, the importance of these being measurable, concrete indications to check the quality of healthcare providers, and secondly, the need for lists to be relatively small. As a result, the measures were updated by the HHT Board (CLS, SDG and EB) to be patient-focused, fewer, easier to measure, and avoid approaches still under discussion in HHT Expert Centres and /or where there was no consensus.

On 14/5/2016 the summary list was circulated to all clinicians with a comment that these had to be (a) universally applicable, (b) non controversial, (c) robust to emerging new evidence, (d) readily achievable as demonstrators of HCPs compliance with good practice.

The email exchanges continued and included the HHT Patient WG that took formal shape between 13-26 May 2016. They requested inclusion of cerebral AVM screening and prenatal diagnoses, leading to internal discussions regarding the progress of cerebral AVM screening through the process, and the need to avoid controversial topics for this particular set of universally-applicable outcome measures.

All agreed, and the list of 5 outcome measures were embedded in the formal June 2016 submission of the ERN Network Application, and the Healthcare Providers' applications.

The manuscript text was developed through 2017-2018.

**Table S1**
